# Predicting leaf nitrogen content in wolfberry trees by hyperspectral transformation and machine learning for precision agriculture

**DOI:** 10.1371/journal.pone.0306851

**Published:** 2024-09-26

**Authors:** Yongmei Li, Hao Wang, Hongli Zhao, Ligen Zhang

**Affiliations:** 1 School of Civil and Hydraulic Engineering, NingXia university, Yinchuan, Ningxia, People’s Republic of China; 2 Institute of Agricultural Economy and Information Technology, Ningxia Academy of Agriculture and Forestry Sciences, Yinchuan, Ningxia, People’s Republic of China; 3 Department of Water Resources, China Institute of Water Resources and Hydropower Research, Beijing, People’s Republic of China; 4 State Key Laboratory of Simulation and Regulation of Water Cycle in River Basin, Beijing, People’s Republic of China; 5 Ningxia Academy of building Research Co., Ltd, Yinchuan, Ningxia, People’s Republic of China; Universidade Federal de Uberlandia, BRAZIL

## Abstract

Leaf nitrogen content (LNC) is an important indicator for scientific diagnosis of the nutrition status of crops. It plays an important role in the growth, yield and quality of wolfberry. This study aimed to develop new spectral indices (NSIs) and constructed machine learning regression (MLR) models for predicting wolfberry tree LNC. By utilizing four smoothing methods and five mathematic transformation methods, we obtained the original spectral dataset and five spectral transformation datasets for quantitative analysis and model establishment. Subsequently, published vegetation indices (PVIs) were acquired, sensitive wavelengths (SWs) were screened and NSIs were calculated based on SWs. Then MLR models were constructed by combining NSIs from six spectral datasets with three machine learning algorithms. Finally, a comparison was made among the MLR models. The study indicated that the application of mathematical transformation highlighted the differences in spectra, the square root, first-derivative and second-derivative transformation improved the prediction accuracy of MLR models constructed based on NSIs (MLR-NSIs models). However, these transformations had little impact on improving the prediction ability of MLR models constructed based on PVIs (MLR-PVIs models). Additionally, The optimal model for predicting the LNC of wolfberry tree was obtained by using the Random Forest (RF) algorithm combined with NSIs developed by first-derivative transformation spectra. The determination coefficient of validation (R_v_^2^) and ratio of percentage deviation (RPD) was 0.71 and 1.90, respectively. In conclusion, this study has demonstrated that the combination of hyperspectral transformation and machine learning is useful for improving the accuracy of LNC estimation in wolfberry trees.

## Introduction

Wolfberry is an important economic crop with the most local characteristics and advantages, and it becomes a pillar industry for local ecological governance and poverty alleviation in Ningxia Province, China. In 2021, the area of wolfberry trees accounted for about 28666.7 ha in Ningxia Province, with a production of 25 billion RMB. By 2027, Ningxia plans to double the cultivated area and output value of wolfberry. Therefore, the high-quality development of wolfberry in Ningxia province receives much attention.

Nitrogen is an essential element for plant growth [1,2] and plays an important role in crop yield and quality [3]. Also, it is the mineral nutrient most commonly used in wolfberry orchard fertilization. Due to the high economic benefits, farmers usually use excessive chemical fertilizers in the planting of wolfberry. However, excessive nitrogen not only increases agricultural costs and pollutes the environment and underground water [4,5] but also affects crop yield and quality [6,7]. Currently, precision water and fertilizer integration technologies are imple-mented to reduce economic costs,mitigate environmental impacts, and promote the high-quality and sustainable development of the wolfberry industry. Evaluating wolfberry tree LNC is important and necessary to provide a scientific reference for better nitrogen nutrition administration by using water and fertilizer integration technologies.

Traditional methods commonly used for diagnosing crop nitrogen status include appearance diagnostic and chemical diagnostic methods. The appearance diagnostic methods involve assessing plant leaf color, growth patterns and symptoms. However, these method are subjective and prone to inaccuracy due to its reliance on visual identification of characteristic symptoms by individuals with varying levels of experience [8]. Some chemical diagnostic methods of laboratory determination based on field sampling are widely used to analyze the nitrogen or photosynthetic pigment content of plants, and they are proven to be accurate and repeatable, but these methods are destructive, time-consuming and laborious [9,10]. Additionally, traditional nitrogen estimates provide limited information due to sampling being restricted to a small number of sites within a given field [11,12]. Therefore, efficient alternatives are necessary. Hyperspectral remote sensing technology provides a new way to solve this problem owing to its high spectral resolution, simplicity, effectiveness, and non-destructiveness. Near-earth remote sensing offeres a reliable method for data collection in precision agriculture through spectral monitoring [13].

There have been many studies on hyperspectral techniques for predicting nitrogen and chlorophyll levels in plants. such as wheat [14–17], rice [18,19], maize [20–22], apple trees [23] and olive trees [11]. Recently, the combination of hyperspectral feature parameters derived from transformation spectra and machine learning algorithms is being utilized to develop predictive models with high accuracy [24,25]. Previous studies have shown the feasibility of using hyperspectral remote sensing for monitoring plant nutrition, and hyperspectral remote sensing technology has become a major development trend in monitoring the nitrogen content of crops[26]. Despite the wide literature on nitrogen estimation by spectral measurements, very few studies are related to this work.

LNC is the most important indicator for evaluating the growth status of wolfberry trees. The timely and effective observation of nitrogen level is essential for guiding fertilization and controlling the growth, yield and quality of wolfberry [27,28**]**. However, to the best of our knowledge, as for the wolfberry tree, there is no study on predicting wolfberry LNC by hyperspectral data. In this study, our aims are to develop some NSIs and to construct the models for predicting wolfberry tree LNC by combining transformation hyperspectral data with machine learning algorithms. To achieve this goal, the following sub-objectives need to be addressed:

Testing four different smoothing methods (Savitzky–Golay smoothing, fast Fourier transform, continuous wavelet transform and nine-point weighted moving average method) to reduce the noise of the reflectance.

Using five mathematical transformations (reciprocal, logarithm, square root, first-derivative and second-derivative) to obtain spectral transformation datasets and analyzing the potential of the spectral datasets for estimating the LNC in Wolfberry trees.

Applying a wavelength combination-based method (difference, ratio and normalization) to develop new spectral indices (NSIs) that improve the fit of published vegetation indices (PVIs) to the LNC in wolfberry trees.

Comparing the predictive power for wolfberry tree LNC of three machine learning regression (MLR) models constructed based on PVIs and NSIs.

## Materials and methods

### Study area

The fieldwork was conducted from June to August 2019 in a wolfberry orchard located in Xinbu town, Zhongning County (105°26’~106°7’E, 37°9’~37°50’N), Ningxia Province, China. Its climate is continental arid and semi-arid in the north temperate zone, with an average annual temperature of 11.3°C and mean annual precipitation of 213 mm. The frost-free season is from 200 d to 205 d, the total annual sunshine is about 3476 h, and the average wind speed is 2 m/second. The terrain is hilly mixed with some alluvial valley plains, with an average altitude of 1140~1600 meters.

The study site has an area of 6.67 ha. According to the Chinese Soil Taxonomy System (1992), the soil type is mainly sandy loam soil. A drip irrigation system is used in this study site with water and nitrogen fertilizer integra-tion. All the wolfberry trees are planted with a fixed spacing in the spring of 2015. The row spacing and the plant spacing of the wolfberry trees are 3 m and 1m, respectively.

### Sample collection

Ningqi No.7, one of the most widely cultivated varieties in Ningxia Province, was selected as the research object in this study. 52 healthy wolfberry trees were chosen as samples from the study area. To determine the nutritional status of wolfberry trees, a leaf analysis was conducted. Due to the variations in branch growth direction of wolfberry trees, leaves were randomly collected from new shoots in the east, south, west and north of each tree to represent the overall nutritional status. Each sample consisted of 100 g of healthy leaves collected from four orientations, with approximately 25 g of leaves obtained from each direction. A total of 52 leaf samples were gathered. Then, the leaves were placed in bags. The bags were sealed, labeled, placed in small mobile refrigerators filled with ice packs and brought back to the laboratory.

### Leaf spectral reflectance measurement

The spectral reflectance was measured using an ASD FieldSpec 3 portable field spectrometer (Analytical Spectral Devices Inc., Boulder, USA) in a darkroom. The leaves arranged in a pile on a black mat with a thickness of 1 cm. A 50-Watt halogen lamp served as the light source, entering at a constant angle (45° from horizontal) and position-ed 30 cm away from the center of the leaf sample. The probe, with a field angle of 25°, was placed perpendicular to the surface of the leaf sample at a distance of 10 cm. The spectral reflectance for every leaf sample was determined as the mean of 10 sample lines. Additionally, a white reference panel was utilized for reflectance correction to ac-count for fluctuations in the light source.

### LNC measurement

After measuring the spectra, all leaf samples were immediately placed in an oven at 104°C for 30 min, dried to a constant weight at 70°C, then ground into powder and passed through a 0.25-mm screen. Subsequently, 0.1 g of powder extracted from each sample were used to determine nitrogen content. The nitrogen content was determined using a Kjeltec™ 8400 nitrogen analyser (FOSS, Hillerød, Denmark). In total, 52 nitrogen data were obtained. The foliar analyses (n = 52) indicated that LNC varied between 3.53% and 4.46%, with an average concentration of 4.098±0.224. The total samples are divided into a calibration set of 35 samples and a verification set of 17 samples randomly. The statistical results are shown in Table 1.

**Table 1:** The nitrogen content of the leaf samples. The "Total" represents the collection of all wolfberry leaf samples (n = 52) in this study. The "Calibration set" and "Validation set" represent wolfberry samples used for model construction and model validation respectively.

Sample	n	Maximum	Minimum	Mean	Median	standard deviation	variance	Coefficient of variation
		g kg^-1^						
Total	52	4.46	3.53	4.098	4.155	0.224	0.050	0.055
Calibration set	35	4.34	3.53	4.090	4.16	0.222	0.049	0.054
Validation set	17	4.46	3.77	4.114	4.11	0.236	0.056	0.057

### Spectral data pre-processing

In the signal acquisition process, spectra are often prone to different interferences due to the external environ-ment, the instrument’s error, the structural and physical properties of the sample [11,29]. Therefore, to avoid the undesired influence on measured spectra, noise reduction is indispensable [30]. The common method to eliminate data noise is spectral smoothness. In this study, the raw data were smoothed by using the following methods: Savitzky–Golay Smoothing(S-G) [31], Fast Fourier Transform (FFT) [32], Continuous Wavelet Transform (CWT) [33] and Nine-point Weighted Moving Average (NWMA) [34]. The smoothed spectral data were labeled as the original spectra (R).

It was impossible to quantitatively judge the quality of smoothing methods by visual discrimination, so the spectral smoothness index (SSI) [35] was adopted in this study to evaluate the smoothness effect of spectral curves. The calculation of SSI is shown in Formula (1):

$$SSI=\frac{\sum {\left(\lambda_{i+1}^{\prime }-\lambda_{i}^{\prime }\right)}^{2}}{\sum {\left(\lambda_{i+1}-\lambda_{i}\right)}^{2}}$$ (1)

where SSI represents the spectral smoothness index; *λ*_*i*_ and $\lambda_{i}^{\prime }$ represent the spectrum of wavelength *i* before spectral smoothness and after spectral smoothness, respectively. The smaller the SSI, the smoother the whole spectral curve.

Mathematical transformation is one of the most effective analytic techniques for hyperspectral data [36]. It can highlight spectral features [37] and extract vegetation biochemical information [38,39]. In this study, five types of mathematical transformations including reciprocal(1/R), logarithmic(logR), square root(R^1/2^), the first-derivative(R′) and the second-derivative (R′′) transformations were performed on the original spectra and the corresponding spectral datasets were obtained. In the follow-up study, the original spectral dataset and five spectral transformation datasets were used as the basis datasets for quantitative analysis and model establishment.

### Hyperspectral features

To explore the spectral indices applicable for LNC prediction of wolfberry tree, we gathered as many published vegetation indices (PVIs) as possible, while also developing new spectral indices (NSIs). Table 2 summarizes the vegetation indices used by other studies to determine the nitrogen status of crops.

**Table 2:** Published vegetation indices used by other studies.

Vegetation Indices	Calculation formula	Reference
RVI	R_800_/R_670_	[40]
NDVI	(R_800_-R_670_)/(R_800_+R_670_)	[41]
VOG1	R_740_/R_720_	[42]
VOG3	(R_734_-R_747_)/(R_715_+R_720_)	[43]
MTCI	(R_750_-R_710_)/(R_710_-R_680_)	[44]
GM	R_750_/R_700_	[45]
SR705	R_750_/R_705_	[46]
mSR705	(R_750_-R_445_)/(R_705_-R_445_)	[47]
ND705	(R_750_-R_705_)/(R_750_+R_705_)	[46]
mND705	(R_750_-R_705_)/(R_750_+R_705_-2R_445_)	[47]
CI_red edge_	R_750_/R_720_-1	[48]
NDVI_gb_	(R_573_-R_440_)/(R_573_+R_440_)	[49]
VI_opt_	(1+0.45)(R_800_^2^+1)/(R_670_+0.45)	[50]
NDRE	(R_790_-R_720_)/(R_790_+R_720_)	[51]
MCARI	(R_700_-R_670_-0.2(R_700_-R_550_))(R_700_/R_670_)	[52]
MTVI2	1.5(1.2(R_800_-R_550_)-2.5(R_670_-R_550_))/sqrt((2R_800_+1)^2^-(6R_800_-5sqrt(R_670_))-0.5)	[53]
CVI1	MCARI/MTVI2	[54]
PPR	(R_550_-R_450_)/(R_550_+R_450_)	[55]
PRI	(R_570_-R_531_)/(R_570_+R_531_)	[56]
GNDVI	(R_750_-R_550_)/(R_750_+R_550_)	[57]
NPCI	(R_430_-R_680_)/(R_430+_R_680_)	[58]
NRI	(R_570_-R_670_)/(R_570+_R_670_)	[59]
SIPI	(R_810_-R_460_)/(R_810_+R_460_)	[60]
TVI	0.5[120(R_750_-R_550_)-200(R_670_-R_550_)]	[61]
VARI	(R_550_-R_670_)/(R_550_+R_670_-R_445_)	[62]
TGI	-0.5[(λ_670_-λ_480_)(R_670_-R_550_)-(λ_670_-λ_550_))(R_670_-R_480_)]	[63]
TCARI	3((R_700_- R_670_)-0.2(R_700_-R_550_)(R_700_/R_670_))	[64]
OSAVI	1.16(R_800_-R_670_)/(R_800_+R_670_+0.16)	[65]
CVI2	TCARI/OSAVI	[64]
DCNI	(R_720_-R_700_)/(R_700_-R_670_)/(R_720_-R_670_+0.03)	[15]
SR_550,670_	R_550_/R_670_	[66]
SR_780,550_	R_780_/R_550_	[66]
SR_780,670_	R_780_/R_670_	[66]
NDI_780,670_	(R_780_−R_670_)/(R_780_+R_670_)	[66]
REP	(R_670_+R_780_)/2	[67]

Mltivariable stepwise regression (MSR) is a simple and effective method to avoid multicollinearity among factors and screen characteristic variables [68]. Therefore, it was employed to screen sensitive wavelengths (SWs) that can represent the LNC of wolfberry tree. The screening SWs was performed through forward selection by using the Scientific Platform Serving for Statistics Professional (SPSSPRO) in china. The reflectance values of the original, reciprocal, logarithmic, square root, first-derivative, and second-derivative spectra within the range of 350 nm to 2500 nm were considered as independent variables, with LNC being the dependent variable. The significance level of the selected variable was set to 0.01 and the excluded variable was set to 0.05. Subsequently, new spectral indices (NSIs) were calculated based on the screened SWs. These NSIs included difference spectral index (DSI), ratio spectral index (RSI) and normalized difference spectral index (NDSI). The NSIs are calculated as follows:

$$DSI=R_{\lambda i}-R_{\lambda j}$$ (2)

$$RSI=R_{\lambda i}/R_{\lambda j}$$ (3)

$$NDSI=(R_{\lambda i}-R_{\lambda j})/({R_{\lambda i}+R}_{\lambda j})$$ (4)

where R represents the spectral reflectance, and λi represents the wavelength.

### Machine learning regression model

#### Construction of the machine learning regression model

In this study, the hyperspectral feature was used as the independent variable, and three machine learning algori-thms, namely, Adaptive Boosting (AdaBoost), Extra Trees(ET) and Random Forest (RF) were used to construct LNC prediction models.

AdaBoost is an iterative algorithm that combines multiple weak classifiers (typically single decision stumps) to form a strong classifier [69]. Weak classifiers usually refere to classifiers with slightly better generalization ability than random guessing, while strong classifiers have a generalization ability very close to the true value. In AdaBoost, each round of iteration adjusts sample weights based on the previous learning results so that the misclassified samples receive more attention in the subsequent round. Simultaneously, the algorithm assign a weight to each weak classifier based on its performance on the current training set (giving high weight to classifiers with low rates and low weight to classifiers with high rates) [69,70]. All weak classifiers are combined in a weighted manner to form the final strong classifier. RF is an ensemble learning algorithm that consists of multiple decision trees. It is often used in classification and regression problems. And it produces good results for both linear and nonlinear data [71]. ET is a relatively new machine-learning algorithm and is developed as an extension of the random forest algorithm. It employs the same principle as RF and uses a random subset of features to train each base estimator [72]. Meanwhile, it uses the whole training dataset to train each regression tree. In contrast, RF uses a bootstrap replica to train the model [72].

#### Robustness and model performance analysis

The application of multiple evaluation indicators can provide insights into the overall accuracy, variability, and explanatory power of the model. In this study, the determination coefficient (R^2^), the root mean square error (RMSE), mean absolute error (MAE) and ratio of percentage deviation (RPD) were used to evaluate the robustness of the prediction models. R^2^ is a statistical measure of the correlation between variables, with values ranging from 0 to 1[73]. The larger R^2^ was, the better the fit between the predicted values and the observed values. RMSE is the square root of Mean Square Error (MSE) which is the average of the squared differences between the predicted values and the observed values [73]. MAE measures the average absolute difference between predicted values and observed values [74]. The smaller RMSE and MAE are indicating that predicted values are closer to observed value. RPD is defined as a ratio of the standard deviation (SD) of the laboratory measured data to the root-mean-square error (RMSE) of prediction [75,76]. Therefore, when R^2^ approaches 1 while RMSE and MAE approach 0, it indicates better predictive ability for a model. An RPD value less than 1.4 suggests poor prediction quality; an RPD value between 1.4 and 1.8 indicates fair prediction suitable for assessment; an RPD value between 1.8 and 2.0 suggests good prediction where quantitative predictions are possible; an RPD value larger than 2.0 indicates very good ability to estimate samples[77]. The robustness of the prediction models was evaluated by:

determination coefficient of calibration (R_c_^2^);

determination coefficient of validation (R_v_^2^);

root mean square error of calibration (RMSE _c_);

root mean square error of validation (RMSE_v_);

mean absolute error of calibration (MAE_c_);

mean absolute error of validation (MAE_v_);

ratio of percentage deviation (RPD).

The R^2^, RMSE, MAE and RPD are given as follows:

$$R^{2}={\left[\frac{\sum_{i=1}^{n}\left(X_{i}-\bar{X})(Y_{i}-\bar{Y}\right)}{\sqrt{\sum_{i=1}^{n}{\left(X_{i}-\bar{X}\right)}^{2}}\sum_{i=1}^{n}{\left(Y_{i}-\bar{Y}\right)}^{2}}\right]}^{2}$$ (5)

$$RMSE=\sqrt{\frac{\sum_{i=1}^{n}{\left(X_{i}-Y_{i}\right)}^{2}}{n}}$$ (6)

$$MAE=\frac{1}{n}\sum_{i=1}^{n}\left|X_{i}-Y_{i}\right|$$ (7)

$$RPD=\frac{SD}{RMSE}$$ (8)

where *X*_*i*_ and *Y*_*i*_ represent the estimated and measured values, respectively. n represents the number of samples. $\bar{X}$ and $\bar{Y}$ represent the average of estimated and measured values, respectively. SD and RMSE are the standard deviation and root mean square error of the validation set, respectively.

## Results

### Spectral data preprocessing

The raw spectra of 52 wolfberry leaf samples are smoothed by using S-G, FFT, CWT and NWMA methods and the mean reflectance of four smoothed spectra is calculated, respectively. The four smoothing methods have similar effects on spectral curves. It is difficult to determine which smoothing method worked best by visual discrimination. Therefore, SSI is used to evaluate the smoothing quality. FFT has the smallest SSI, so the spectra smoothed by FFT are selected as the original spectra(R) in this study (S1 Fig).

**Fig 1:**
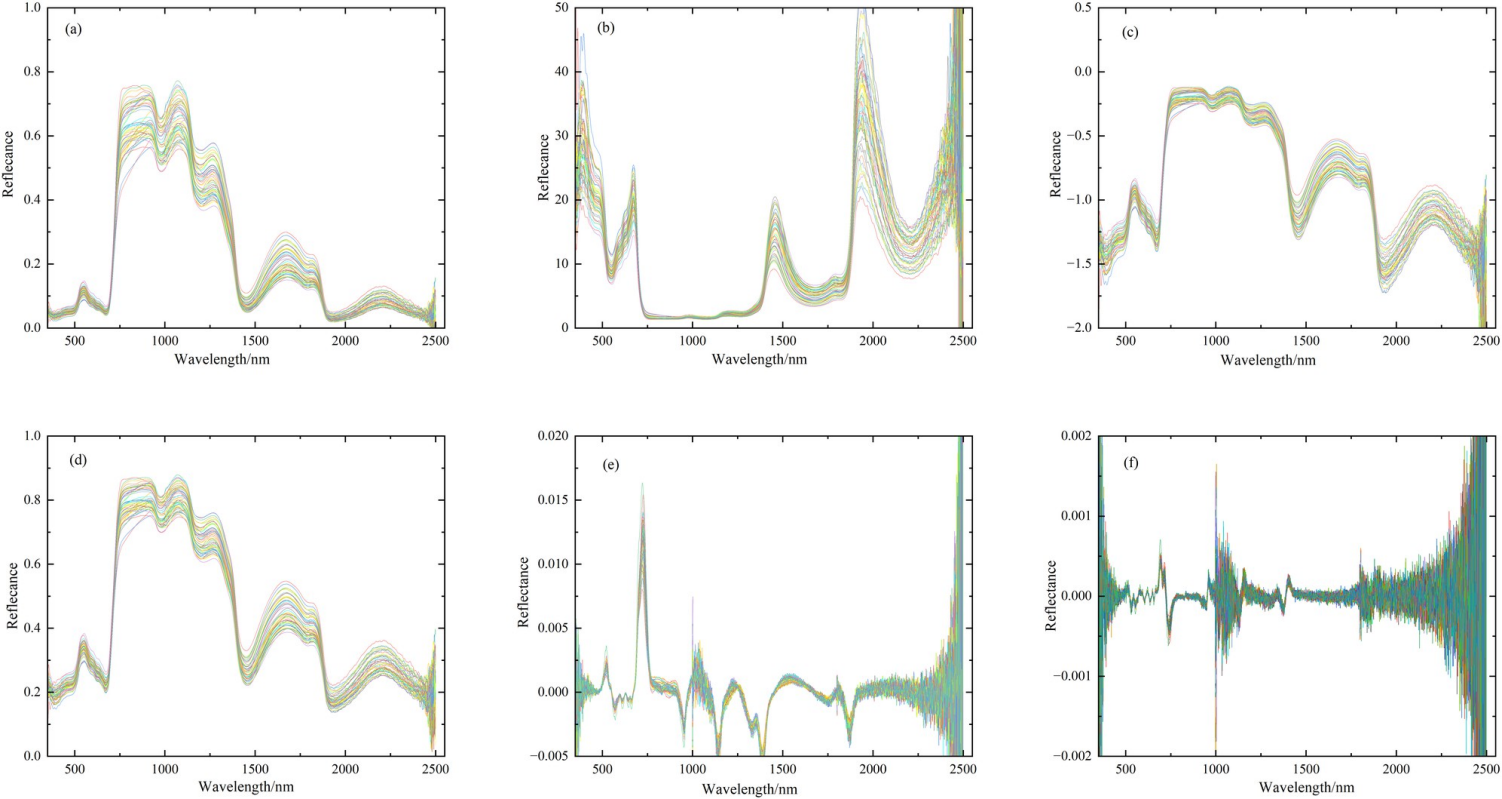
The curves of the original spectra and five types of transformation spectra. Each color line represents a sample reflectance curve, with a total of 52 samples. (a) represents the original spectra (R); (b), (c), (d), (e) and (f) represent the reciprocal (1/R), logarithmic (logR), square root (R^1/2^), first-derivative (R’) and second-derivative spectra (R′′), respectively.

Six types of spectral curves (S1 Fig) are shown in Fig 1. Compared with the original spectra, reciprocal, logarithmic and square root transformations all enhance the difference of spectral reflectance in the VIS and in the SWIR region to varying degrees. Meanwhile, the first-derivative and second-derivative transformation effectively improve the resolution of the overlapping spectra, so the difference at the red-edge position (680~760nm) is increased. Therefore, the mathematical transformation can improve the spectral features to a certain extent, which helps to distinguish the impact of the difference in nitrogen content on the spectra.

### Hyperspectral features analysis

#### Relationship of PVIs to LNC

**Fig 2:**
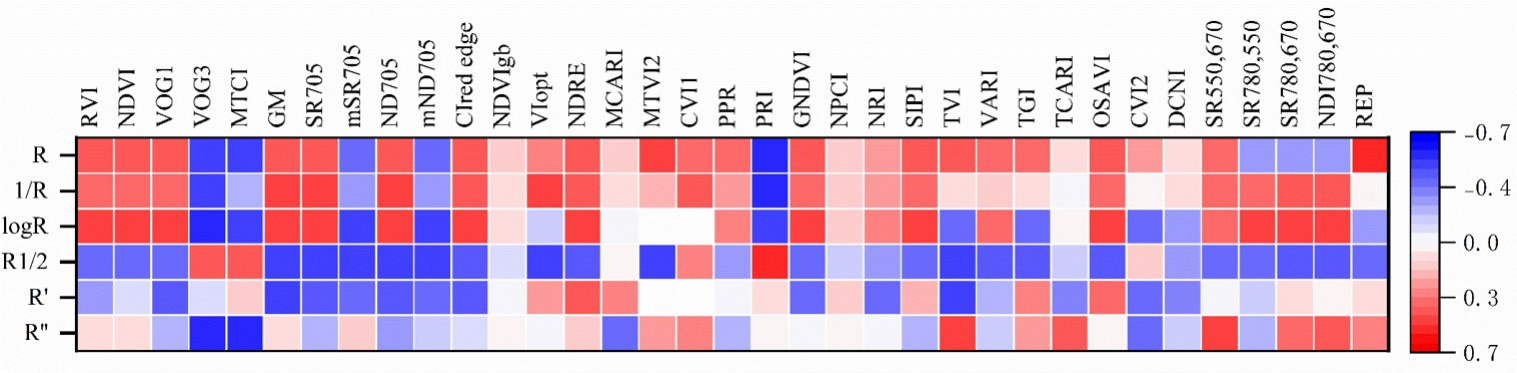
The correlation coefficient between PVIs with LNC in different spectral datasets. R, 1/R, logR, R^1/2^, R′ and R′′ represent the original spectra, reciprocal spectra, logarithmic spectra, square-root, first-derivative and second-derivative spectra, respectively.

Fig 2 represents the correlation coefficient between PVIs and LNC(S2 Fig). In the original spectra, the 24 PVIs (accounting for 69%) pass the 0.01-significance test and are significantly correlated with LNC. The absolute values of the correlation coefficients of PRI, REP, and VOG3 are the largest, which are -0.544,-0.528 and -0.521, respectively. In reciprocal spectra, there are19 PVIs with a significance level of 0.01, accounting for 54% of all PVIs. The absolute values of the correlation coefficients of PRI, VOG3 and GM are the largest, which are -0.535, -0.511 and 0.487, respectively. In the logarithm spectra, 23 PVIs pass the 0.01-significance test, accounting for 66%. The absolute value of the correlation coefficient of VOG3 is the largest, which is -0.525. Followed by PRI and GM, with the correlation coefficients of -0.519 and 0.507, respectively. In the square root spectra, 26 PVIs pass the 0.01-significance test, accounting for 74%. PRI has the largest correlation coefficient of 0.535. The mSR_705_ and mND_705_ have the second highest correlation coefficients, both with value of -0.515. In the first-derivative and second-derivative spectra, 3 and 9 PVIs pass the 0.01-significance test, accounting for 37% and 26%, respecttively. In the first-derivative spectra, the top three PVIs with the absolute value of the correlation coefficients are GM, TVI and CI_red edge_, with values of -0.497, -0.489 and -0.442. In second-derivative spectra, the top three PVIs are VOG3, MTCI and TVI, with values of -0.561, -0.538 and 0.502. Overall, the spectral transformation has little effect on improving the correlation between PVIs and LNC. Only there is the VOG3 in second-derivative spectra whose correlation coefficient is slightly greater than that of the original spectra.

#### Relationship of NSIs to LNC

*Sensive wavelengths selection*. The selected SWs are presented in Table 3. The SWs of the original, reciprocal, logarithmic and square root reflectance are observed at 359 nm in the VIS region and near 770nm in the NIR region. A total of 16 SWs are identified from the first-derivative spectra, including 379nm, 388nm and 449nm distributed in the VIS region, as well as 745 nm and 1128nm distributed in the NIR region and the remaining SWs distributed in the SWIR region. However, the SWs of the second-derivative reflectance don’t appeare in VIS region but are instead distributed in the NIR and SWIR region. Upon comparing the SWs from different spectral datasets, it is found that the first-derivative transform spectra have the higher number of SWs and cover the wider spectral range.

**Table 3:** The sensitive wavelengths selected by MSR. R, 1/R, logR, R^1/2^, R′ and R′′ represent the original spectra, reciprocal spectra, logarithmic spectra, square-root, first-derivative and second-derivative spectra, respectively. The selected sensitive wave-lengths rank according to wavelength.

spectra	Sensitive wavelengths (nm)
R	359, 772
1/R	359, 771
logR	359, 770
R^1/2^	359, 771
R’	379, 388, 449, 745, 1128, 1610, 1945, 2005, 2051, 2073, 2092, 2102, 2109, 2153, 2240, 2442
R′′	759, 774, 787, 1009, 1028, 1147, 1454, 1518, 1585, 1641, 1643, 1796, 1950, 2202, 2359

*Relationship of NSIs to LNC*. NSI was calculated based on the SW_S_ data in Table 3 resulting in a total of 687 NSIs. Correlation analysis between NSIs and LNC was conducted. As indicated in Table 4, the original spectra, the logarithmic spectra and the square-root spectra all show that all NSIs pass the 0.01-significance test. The NSIs with the strongest correlation are NDSI_R(772,359)_, DSI_logR(770,359)_ and DSI_R_^1/2^_(771,359)_, with a correlation coefficient of -0.556, -0.563 and -0.599, respectively. In the reciprocal spectra, RSI_1/R(771,359)_ and NDSI_1/R(771,359)_ pass the 0.01-significance test while DSI_1/R(771,359)_ only passes the 0.05-significance test. In the first-derivative spectra, a total of 51 NSIs are correlated with LNC at a significance level of 0.01 and the top three NSIs are DSIR’_(1128,388)_, DSIR’_(745,449)_ and DSIR’_(1945,745)_, with correlation coefficients of 0.597, -0.582 and 0.573, respectively. In the second-derivative spectra, a total of 29 NVIs passe the 0.01-significance test and the top three NSIs are DSIR’’_(1641,787),_ DSIR’’ _(1641,759)_ and DSIR’’_(1518,759)_, with the correlation coefficients of -0.644, -0.600 and -0.551, respectively.

**Table 4:** The correlation coefficients of all new spectral indices in the original spectra, the reciprocal spectra, logarithmic spectra and square root spectra. And the top three NSIs significantly correlated with LNC in first-derivative spectra and second-derivative spectra. The correlation coefficient refers to the result of correlation analysis between LNC and NSIs calculated by SWs from different spectral datasets. DSI, RSI and NDSI represent the difference spectra index, ratio spectral index and normalized difference spectral index respectively. R, 1/R, logR, R^1/2^, R’ and R′′ represent the original spectra, the reciprocal spectra, logarithmic spectra, square root spectra, first-derivative spectra and second-derivative spectra respectively. The NSIs are listed in descending order of the absolute values of the correlation coefficients.

spectra	NSIs	Correlation Coefficients	spectra	NSIs	Correlation Coefficients
R	NDSI_R(772,359)_	-0.556**	R^1/2^	DSI_R_^1/2^_(771,359)_	-0.599**
	RSI_R(772,359)_	0.554**		NDSI_R_^1/2^_(771,359)_	-0.562**
	DSI_R(772,359)_	-0.467**		RSI_R_^1/2^_(771,359)_	0.560**
1/R	NDSI_1/R(771,359)_	0.556**	R’	DSIR’_(1128,388)_	0.597**
	RSI_1/R(771,359)_	-0.554**		DSIR’_(745,449)_	-0.582**
	DSI_1/R(771,359)_	0.334*		DSIR’_(1945,745)_	0.573**
	DSI_logR(770,359)_	-0.563**	R′′	DSIR’’_(1641,787)_	-0.644**
logR	NDSI_logR(770,359)_	0.501**		DSIR’’_(1641,759)_	-0.600**
	RSI_logR(770,359)_	-0.410**		DSIR’’_(1518,759)_	-0.551**

** and * indicate a significant correlation at the levels of 0.01 and 0.05 respectively.

### The predicting model

#### Machine learning regression models based on PVIs

**Fig 3:**
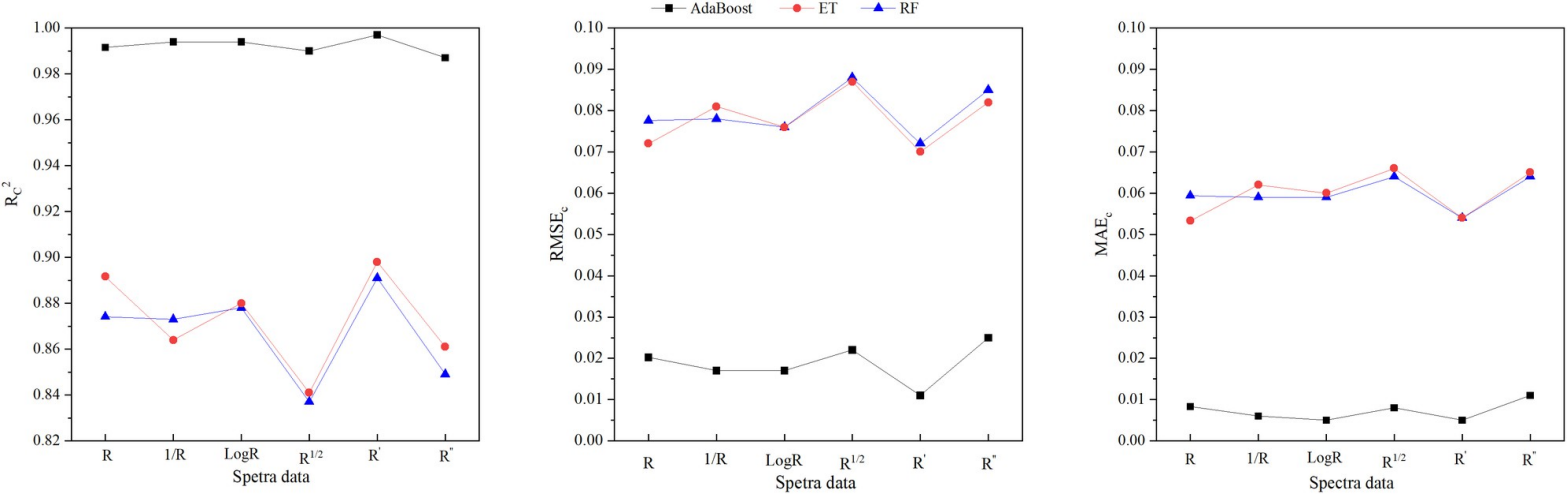
R_c_^2^,RMSE_c_ and MAE_c_ distributions of the machine learning regression models based on PVIs (MLR-PVIs models) in differrent spectral dataset. R_c_2,RMSE_c_ and MAEc represente determination coefficient, root mean square error and mean absolute error of calibration respecttively. R, 1/R, logR, R^1/2^,R′ and R′′ represent original spectra, reciprocal spectra, logarithmic spectra, square root spectra, first-drivative and second-derivative spectra respectively. AdaBoost, ET and RF represent Adaptive Boosting models, Extra Trees models and Random forest models respectively.

To assess the feasibility of using PVIs to monitor wolfberry tree LNC and to evaluate the impact of spectral transformations on the response of PVIs to wolfberry tree LNC change. The top three PVIs significantly correlated with LNC were selected as independent variables to establish machine learning regression models for predicting the LNC of wolfberry trees (S3 Fig). Fig 3 illustrates that among the three types of MLR-PVIs models, the Ada-Boost models exhibites the highest R_c_^2^, with a value exceeding 0.987. The R_c_^2^ values of RF and ET models are also high and similar, ranging from 0.837 to 0.891 and from 0.841 to 0.892, respectively. When considering six types of spectral datasets, it is observed that the R_c_^2^ values for AdaBoost models constructed by using five transformation spectra closely resemble those constructed by using original spectra. However, Except for the first-derivative spectra, the R_c_^2^ values of the ET models constructed by using other transformation spectra are lower than those constructed by using the original spectra. Furthermore, it is only in the case of the first-derivative or logarithmic spectra were utilized that the R_c_^2^ value of the RF models slightly exceed those obtained when utilizing original spectra. The above analysis shows that spectral transformation can hardly improve the prediction ability of the MLR-PVIs model.

**Fig 4:**
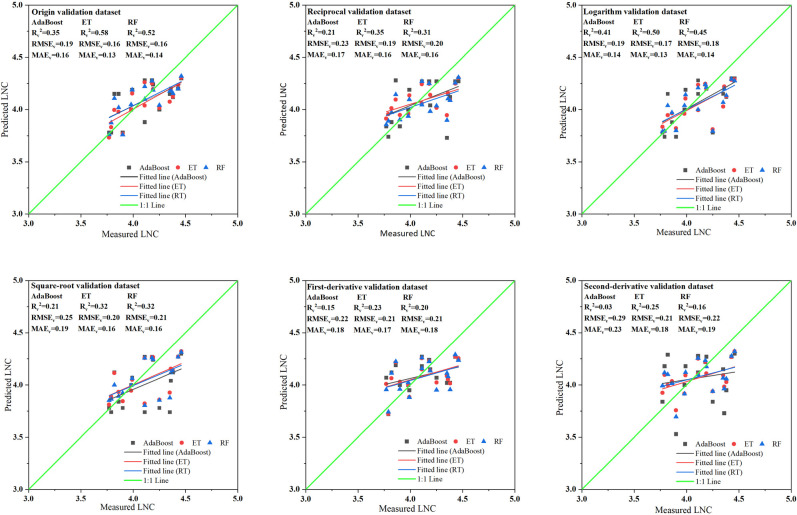
Distribution of measured and predicted values of the machine learning regression models based on PVIs (MLR-PVIs models) in different spectral datasets. AdaBoost, ET and RF represent Adaptive Boosting models, Extra Trees models and Random forest models respectively. R_v_^2^, RMSE_v_ and MAE_v_ represent determination coefficient, root mean square error and mean absolute error of validation respectively.

As shown in Fig 4, the ET models of the three types of MLR-PVIs models exhibit the strongest explanatory ability for LNC with the R_v_^2^ values ranging from 0.23 to 0.58, followed by the RF models with the R_v_^2^ values ranging from 0.16 to 0.52 and then the AdaBoost models with the R_v_^2^ values ranging from 0.03 to 0.35. Among the six types of spectral datasets, both original spectra and logarithmic spectra utilized in constructing MLR-PVIs models show good explanatory ability for LNC change, with the R_v_^2^ values ranging from 0.35 to 0.58 and from 0.41 to 0.50 respectively. However for the other four transformation spectra used in MLR-PVIs models, all show very poor predictive ability for wolfberry tree LNC, and the R_v_^2^ values ranging from 0.03 to 0.35 (S4 Fig). Overall, In the MLR-PVIs models, the ET model constructed by using the original spectra displays a good explanatory ability for LNC change, with the R_v_^2^ of 0.58, the RMSE_c_ of 0.16 and the MAEc of 0.13. Followed by the RF model constructed by using the original spectra and the ET model constructed by using the logarithmic spectra and their R_v_^2^ values are 0.52 and 0.50, respectively. The analysis indicates that spectral transformations had minimal impact on improving the predictive power within MLR-PVIs models.

### Machine learning regression models based on NSIs

**Fig 5:**
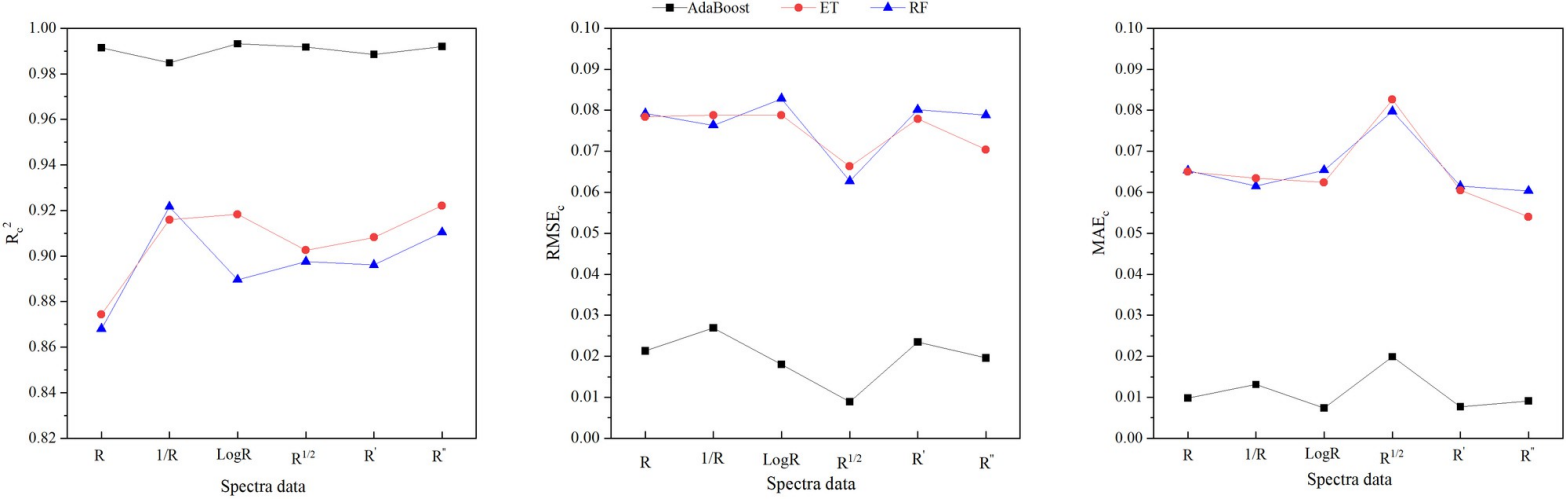
Rc2, RMSEc and MAEc distributions of the machine learning regression models based on NSIs in different spectral dataset. R_c_^2^, RMSE_c_ and MAE_c_ represent determination coefficient, root mean square error and mean absolute error of calibration respectively. R, 1/R, logR, R^1/2^, R′ and R′′ represent original spectra, reciprocal spectra, logarithmic spectra, square root spectra, first-derivative and second-derivative spectra respectively. AdaBoost, ET and RF represent Adaptive Boosting models, Extra Trees models and Random forest models respectively.

The NSIs listed in the Table 4 were used to construct AdBoost, ET and RF models for predicting the wolfberry trees LNC. Fig 5 illustrates that among the three types of MLR-NSIs models, the AdaBoost models exhibit the highest R_c_^2^, with a value exceeding 0.985. The R_c_^2^ values of the RF and ET models are comparable, ranging from 0.868 to 0.922 and from 0.874 to 0.922, respectively (S5 Fig). When considering six types of spectral datasets, it is observed that R_c_^2^ values for AdaBoost models constructed by using five transformation spectra closely resemble those constructed by using original spectra. The R_c_^2^ values of ET and RF models constructed by using the five transformation spectra are greater than those constructed by using the original spectra.

**Fig 6:**
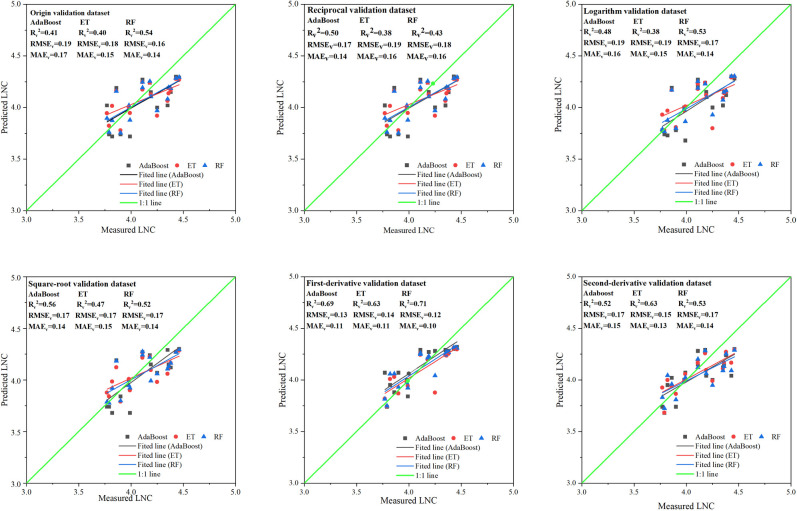
Distribution of measured and predicted values of the machine learning regression models based on NSIs (MLR-NSIs models) in di-fferrent spectral datasets. AdaBoost, ET and RF represent Adaptive Boosting models, Extra Trees model and Random forest models respecttively. R_v_^2^, RMSE_v_ and MAE_v_ represent determination coefficient, root mean square error and mean absolute error of valida-tion respectively.

As illustrated in Fig 6 (S6 Fig), the RF models of the three types of MLR-SIs models obtain the highest R_v_^^2^^, ranging from 0.433 to 0.710, followed by the AdaBoost models with the R_v_^^2^^ values ranging from 0.408 to 0.688, and then the ET models with the R_v_^2^ values ranging from 0.376 to 0.632. Among the six types of spectra datasets, it is found that the first-derivative spectra utilized in constructing MLR-PVIs models showed the strongest explanatory ability for LNC change, with the R_v_^^2^^ values ranging from 0.632 to 0.710. Followed by the second-derivative spectra with R_v_^^2^^ values ranging from 0.517 to 0.628, and then the square root spectra with R_v_^^2^^ values ranging from 0.475 to 0.563. However, for the logarithmic and the reciprocal spectra, it is observed that their R_v_^^2^^ values are lower than those of the original spectra, ranging from 0.377 to 0.534 and ranging from 0.376 to 0.500, respectively. The original spectra used in MLR-NSIs models shows R_v_^^2^^ values between 0.404 and 0.540.

Overall, in terms of MLR-NSIs models, the RF model constructed by using first-derivative spectra demonstrates superior explanatory power with a R_v_^2^ value of 0.710, a RMSE_v_ value of 0.124 and a MAE_v_ value of 0.104. Followed by the AdaBoost model constructed by using the first-derivative spectra, with a R_v_^^2^^ value of 0.688, a RMSE_v_ value of 0.130 and a MAE_v_ value of 0.108. Then the ET model constructed using the first-derivative spectra, with an R_v_^^2^^ value of 0.632, an RMSE_v_ value of 0.141 and a MAE_v_ value of 0.111. Additionally, the ET model constructed by using the second-derivative spectra exhibits good explanatory power as well, with a R_v_^^2^^ value of 0.628. The analysis above indicates that the NSIs developed through spectral transformation can enhance prediction capabilities within MLR-NSIs models.

### Model accuracy comparison

**Fig 7:**
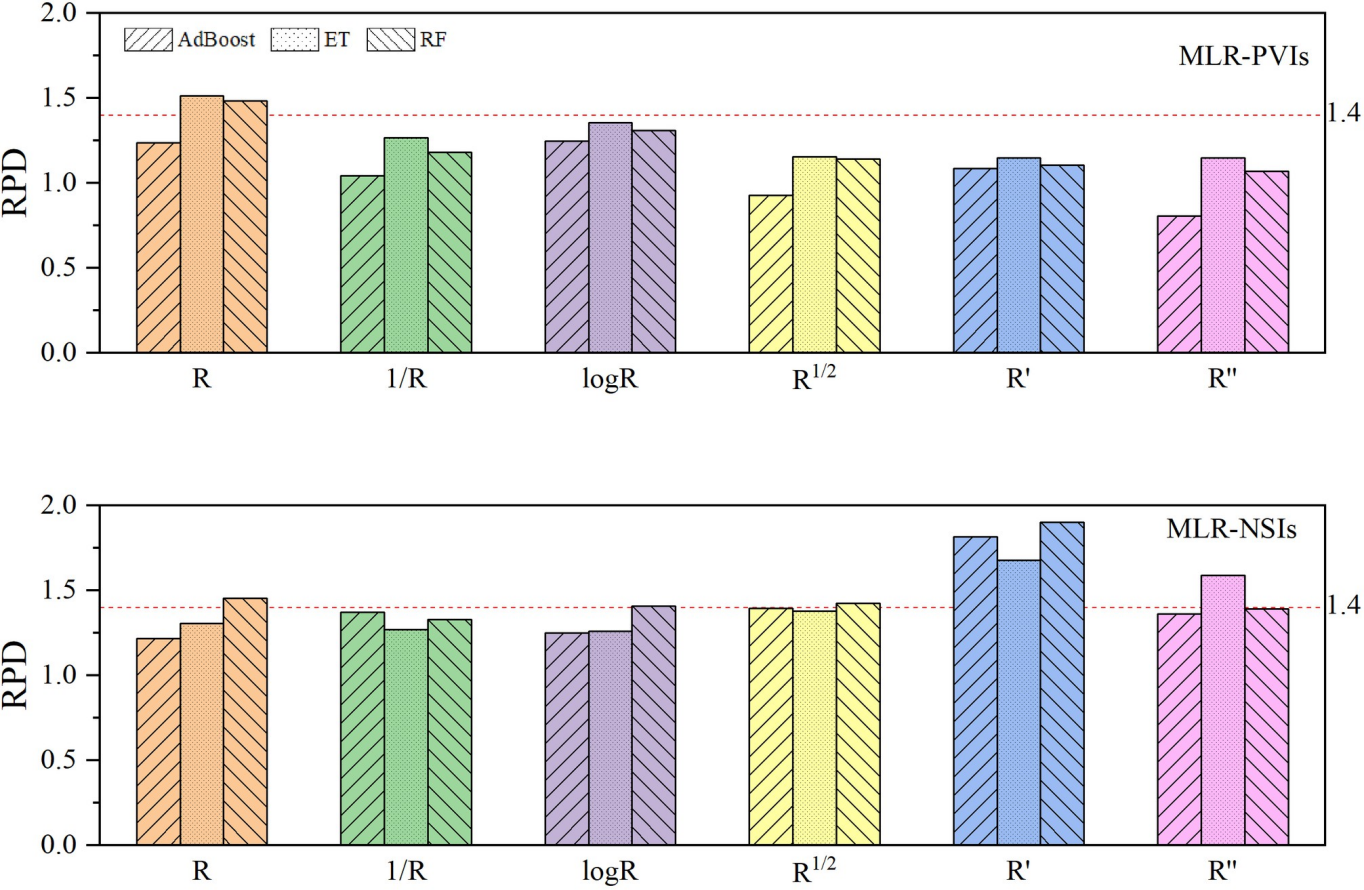
RPD distribution of the MLR-PVIs models and the MLR-NSIs models. The RPD represents the ratio of the standard deviation (SD) of the measured LNC to the root mean square error of predicted LNC. The MLR-PVIs models and the MLR-NSIs models represent the machine learning regression models constructed by using the published vegetation indices and by using the new spectral indices. AdaBoost, ET and RF represent Adaptive Boosting models, Extra Trees models and Random forest models respectively. R, 1/R, logR, R^1/2^, R′ and R′′ represent original spectra, reciprocal spectra, logarithmic spectra, square root spectra, first-derivative and second-derivative spectra respectively.

As shown in Fig 7 (S7 Fig), the RPD values of the MLR-PVIs models constructed by using the five transformation spectra are smaller than those constructed by using the original spectra. Among the five transformation spectra, the logarithmic spectra yield the highest RPD values, with a maximum value of only 1.355. In all MLR-PVIs models, only the ET model and RF model constructed by using the original spectra achieve an RPD value slightly greater than 1.400, with PRD values of 1.513 and 1.483 respectively.

When utilizing the reciprocal transformation spectra, the RPD values of the MLR-NSIs models are less than 1.4. By using the original spectra, the logarithmic spectra and the square root spectra, only the RF models achieve RPD value of slightly larger than 1.4, with RPD values of 1.456, 1.410 and 1.425 respectively. By using the second-derivative spectra, only the RPD value of the ET model was 1.589, all other models have PRD values below 1.400. By using the first-derivative spectra, the RPD values of the RF model, AdBoost model and ET model are1.902, 1.816 and 1.678 respectively. So the MLR-NSIs models constructed by using the first-derivative spectra are outstanding. Overall, the MLR-NSIs models constructed by using the transformation spectra demonstrate better predictive capabilities for wolfberry tree LNC compared to MLR-PVIs models-except for all models of the original spectra and the ET model of the logarithmic spectra.

## Discussion

Hyperspectral data contains a large amount of redundant information due to the relatively few variables that really and effectively control the spectral signatures of vegetation [11,78]. Research has shown that while machine learning can effectively handle complex collinearity problems, the predicttive ability of models are influenced by the varying capabilities of different machine learning algorithms [79]. Additionally, studies have demonstrated that spectral mathematical transformation plays a crucial role in amplifying the differences in spectral reflectance and extracting spectral characteristics [80,81]. In order to identify suitable variables for wolfberry tree LNC and mitigate the impact of collinearity in hyperspectral data on the predictive ability of MLR models, mathematical transformations were applied to the original spectra. Subsequently, SWs were selected from six transformed spectral datasets and NSIs were calculated by combining with the SWs.

In this study, the reciprocal, logarithm and square-root transformation enhance the spectral differences of the VIS and SWIR region. The first-derivative and the-second derivative transformation highlight the subtle spectral differences in the red-edge region. The sensitive spectra located at 359nm, 379nm, 388nm and 449nm in the VIS region are selected.They might be directtly related to the chl a absorption pit at 430 nm and indirectly related to the N content [64,82]. The spectra located at 745nm, 759nm, 774nm, 787nm in the NIR region are selected. These spetra are close to 750nm and are in the limit between red domain of the VIS region and red-edge domain of the NIR region. Several authors selected the hyperspectral reflectance at 750 nm to estimate Chl ab content [83,84]. Therefore, it also could be related to N content. Meanwhile, the selected sensitive wavelengths are found to be consistent with previously published vegetation indices. For example, the sensitive wavelengths from first-derivative spectra (745nm and 449nm) closely match the wavelength of mSR705 (750nm and 450nm) in PVIs [47]. Studies have shown characteristic absorption bands of nitrogen are primarily located in the shortwave infrared (SWIR) region at specific wavelengths such as 1510nm, 1730nm, 1940nm, 1980nm, 2060nm, 2180nm, 2240nm, 2300nm and 2350nm [85]. However, the spectra within the SWIR region are sensitive to leaf water content and the indices which included the 1770nm absorption feature are least affected by leaf water content [86]. The 1128nm, 1610nm, 1945nm, 2051nm, 2073nm, 2092nm, 2153nm and 2240nm selected from first-derivative spectra and the 1641nm, 1643nm, 1796nm, 1950nm, 2202nm, 2359nm selected from second-derivative spectra are located in the SWIR region, which might be related to N and water content [82]. The 1518nm and 1585nm selected from the second-derivative spectra fell within the spectral range of 1500-1600nm, which is a region dominated by absorption features caused by stretching of N-H bonds at 1510nm [82] and this bond is related to the amount of N present in protein [11]. Furthermore, research indicat that employing spectral mathematical transformation can enhance model accuracy by transforming linear relationships between spectral reflectance and observed objects into nonlinear ones [87]. In this study, spectral transformations have little impact on improving the correlation between PVIs and LNC and the predictive power of MLR-PVIs models. However, except for the ET models constructed base on the logarithmic transformation spectra, the response capacity of all the MLR-NSIs models constructed base on the transformation spectra to wolfberry tree LNC are improved compared with the MLR-PVIs models. And compared to the original spectra, the square root, first-derivative and second-derivative transformations have improved the predicttive ability of the MLR-NSIs models.

Many crop nitrogen monitoring models have utilized various types of multiple regressions techniques, including stepwise multiple linear regression [88,89], partial least squares regression [89], and machine learning regression (e.g., support vector machine regression, random forest regression, adaptive boost and bagging.et al) [79,89]. These nonlinear modeling methods can incorporate more spectral information to provide more accurate results. Hyper-spectral evaluation of nitrogen accumulation in winter wheat leaves based on different regression models shows that the model constructed by machine learning can effectively utilize the characteristic information of chlorophyll absorption and improve the estimation accuracy [26]. Ensemble machine learning models (RF、AdBoost and Bagging) have good predictive ability for crop nitrogen nutrition [79]. In this study, the three ensemble machine learning models(AdBoost, ET and RF) constructed based on first-derivative spectra all exhibit strong predictive performance for wolfberry tree LNC. Notably, the RF model constructed using first-derivative spectra demonstrates optimal predictive power. This outcome may also be attributed to the NSIs utilized in the model: DSIR’_(1128,388),_ DSI_R’(745,449)_ and DSI_R’(1945,745)_. These three NSIs encompass spectral features at 449nm in the VIS region, 745nm in the red-edge region, 1128nm in the NIR region and 1945nm in the SWIR region. Spectral information significantly impact a model’s predictive ability and an abundance of such information can enhance it to a certain extent.

Studies have shown that nitrogen content changes with the progression of growth stages [90,91]. Furthermore, it has been indicated that the small sample quantity is not a negligible factor that affects the robustness of the estimation model [26]. As our study was conducted using specific synthetic datasets composed of different growth stages and with limited data (only 52 samples), our models inherently possess limitations in applicability. Addi-tionally, previous studies have mainly focused on farmland with artificially controlled nitrogen levels where high and low N levels were easily obtained [91]. However, variable rate fertilization is rarely carried out in Chinese agriculture, making it challenging to obtain data with large changes in biochemical components as observed in the experiment [91]. Therefore, despite achieving a model with high prediction accuracy in the experiment, its practical application was limited. In this study, wolfberry plantation under normal fertilization conditions of local farmers in Ningxia province was selected as the sample collection area. Consequently, the optimal model for predicting LNC of wolfberry tree obtained in this study holds certain practical reference value for the diagnosis of nitrogen nutrients during specific growth stages of wolfberry (June to August). Additionally, this study combines mathematical transformation with machine learning to provide a spectral selection method and a modeling framework for monitoring wolfberry LNC, which may serve as a useful and valuable tool in nitrogen monitoring and management. To develop accurate, robust and fast model with high reliability, practicability and applicability, the next step should be to validate these models by collecting extensive data for different growth stages, and develop nitrogen prediction models suitable for each growth stages.

## Conclusions

Currently, there is limited research on diagnosing nitrogen levels in wolfberry tree using hyperspectral data. In this study, new spectral indices based on transformation spectra were developed and combined with machine learning (ML) to explore the monitoring of wolfberry tree LNC. The study indicates that spectral transformations enhance the reflectance difference in the VIS and SWIR region, highlight the subtle spectral difference in the red-edge region and improve the accuracy of the MLR-NSIs models. However, they do not have a significant effect on the accuracy of the MLR-PVIs models. In the MLR-PVIs models, it is found that ET model constructed by using the original spectra is optimal, with R_c_^2^, R_v_^2^ and RPD values of 0.892, 0.576 and 1.510 respectively. In contrast, in the MLR-NSIs models, the RF model constructed by using the first-derivative spectra datasets is optimal for predicting wolfberry LNC, with R_c_^2^, R_v_^2^ and RPD values of 0.90, 0.71 and 1.90 respectively. Therefore, although it is feasible to use PVIs to predict wolfberry LNC, the MLR-PVIs models perform worse than the MLR-NSIs model. This study combines spectral transformation with machine learning algorithms to identify a set of NSIs and a machine learning model suitable for detecting wolfberry tree LNC. The set of NSIs are DSIR’_(1128,388)_, DSIR’_(745,449)_ and DSIR’_(1945,745)_ developed by using first-derivative spectral data. This model is a RF model constructed by using this set of NSIs.

## Supporting information

S1 Fig Spectral dataset. The original spectra are derived from the rapid Fourier transform of the raw spectral data.
(XLSX)

S2 Fig Correlation coefficient. PVIs represents published vegetation indices. Origin-PVIs,reciprocal-PVIs, logarithm-PVIs, square root-PVIs,FD-PVIs, and SD-PVIs represents published vegetation indices calculated from the original spectra, reciprocal spectra, logarithm spectra, square-root spectra, first-derivative spectra and second-derivative spectra datasets, respectively.
(XLSX)

S3 Fig Determination coefficient, root mean square error and mean absolute error of calibration of the machine learning regression models based on published vegetation indices. R_c_^2^, RMSE_c_ and MAE_c_ represent determination coefficient, root mean square error and mean absolute error of calibration respectively. R, 1/R, logR, R^1/2^, R′ and R′′ represent original spectra, reciprocal spectra, logarithmic spectra, square root spectra, first-drivative and second-derivative spectra respectively. AdaBoost, ET and RF represent Adaptive Boosting models, Extra Trees models and Random forest models respectively.
(XLSX)

S4 Fig Determination coefficient, root mean square error and mean absolute error of validation of the machine learning regression models based on published vegetation indices. R_v_^2^, RMSE_v_ and MAE_v_ represent determination coefficient, root mean square error and mean absolute error of validation respectively.
(XLSX)

S5 Fig Determination coefficient, root mean square error and mean absolute error of calibration of the machine learning regression models based on new spectral indices. R_c_^2^, RMSE_c_ and MAE_c_ represent determination coefficient, root mean square error and mean absolute error of calibration respectively.
(XLSX)

S6 Fig Determination coefficient, root mean square error and mean absolute error of validation of the machine learning regression models based on new spectral indices. R_v_^2^, RMSE_v_ and MAE_v_ represent determination coefficient, root mean square error and mean absolute error of validation respectively.
(XLSX)

S7 Fig Ratio of percentage deviation of the machine learning regression models. MLR-PVIs models and the MLR-NSIs models are the machine learning regression models based on published vegetation indices and new spectral indices, respectively.
(XLSX)
